# Direct medical cost of first-ever acute ischemic stroke in malaysia: a retrospective cohort study

**DOI:** 10.1038/s41598-025-07026-1

**Published:** 2025-07-02

**Authors:** Mustapha Mohammed, Hadzliana Zainal, Siew Chin Ong, Balamurugan Tangiisuran, Fatimatuzzahra Abdul Aziz, Abubakar Sha’aban, Usman Abubakar, Norsima Nazifah Sidek, Irene Looi, Zariah A. Aziz

**Affiliations:** 1 Biomedical Research Center, QU Health, Qatar University, Doha, Qatar; 2 School of Pharmaceutical Sciences, Universiti Sains Malaysia, Pulau Pinang, Malaysia; 3 Health and Care Research Wales Evidence Centre, Cardiff University, Cardiff, Wales United Kingdom; 4 Department of Clinical Pharmacy and Practice, College of Pharmacy, QU Health, Qatar University, Doha, Qatar; 5 Clinical Research Centre, Hospital Sultanah Nur Zahirah, Kuala Terengganu, Terengganu Malaysia; 6 Clinical Research Centre, Hospital Seberang Jaya, Permatang Pauh, Seberang Jaya, Pulau Pinang, Malaysia; 7 Clinical Research Centre, Hospital Sultanah Nur Zahirah, Kuala Terengganu, Terengganu, Malaysia

**Keywords:** Ischemic stroke, Cost-of-illness, Functional disability, Modified Rankin scale, Registry, Malaysia, Health care, Neurology

## Abstract

Understanding the economic impact of first-ever stroke in a multiethnic population like Malaysia is essential for informed resource allocation. This study aimed to evaluate the direct medical costs associated with first-ever ischemic stroke in Malaysia. The study was a retrospective cohort study that estimated the inpatient direct medical costs of first-ever ischemic stroke in Malaysia. The study involved 122 adult patients managed at Hospital Sultanah Nur Zahirah, Terengganu (HSNZ), enrolled in the Malaysian National Stroke Registry (NSR) from 2009 to 2020. The mean ± standard deviation (SD) for the patients’ age was 61.0 ± 10.9 years, the length of stay (LOS) was 4.5 ± 3.5 days, and the modified Rankin scale (mRS) score was 3.0 ± 1.1. Most patients experienced functional disability (poor functional status, mRS ≥ 3) and incurred an average (SD) treatment cost of MYR 1,970.7 ± 1,385.8, primarily attributed to hospital admissions and radiology expenses. The medical costs were significantly lower in patients with good functional status [mRS < 3 (*p* = 0.002)] and shorter LOS (*p* < 0.001), but higher in patients with the partial anterior circulation infarct (PACI) stroke subtype (*p* < 0.001). Additionally, patients with good functional status incurred significantly lower costs for admission and medications (*p* < 0.001). In conclusion, the inpatient direct medical cost of first-ever ischemic stroke in Malaysia is substantial and is influenced by stroke subtypes, length of stay, risk factors, and functional status. Strategies to reduce the length of stay, comorbidities, and functional status can potentially reduce the economic burden of the first-ever acute ischemic stroke. These findings are crucial for guiding the optimal allocation of resources for stroke care.

## Introduction

Stroke is a leading cause of death and functional disability worldwide, with an attributed substantial economic burden^1^. Stroke is a major public health issue, and effective management of stroke is essential to reduce its impact on the health system, individuals and society^2^. Stroke accounts for about 34% of all healthcare spending worldwide^3^. Ischemic stroke is the most common stroke, accounting for approximately 85% of all stroke cases^4^. Managing ischemic stroke requires prompt diagnosis, timely thrombolytic therapy or mechanical thrombectomy administration, and effective post-stroke care^5^. Inpatient care is an essential component of stroke management, accounting for a significant proportion of the total cost of stroke care^6^. Over 70% of the first-year costs of stroke care are accounted for by inpatient medical costs for acute stroke^7^. Despite the higher cost of medical care, patients with moderate disabilities after stroke benefit more from treatment and rehabilitation than those with severe or mild disabilities^8^. Moreover, the costs of stroke care typically increase due to comorbid conditions, including ischemic heart disease and atrial fibrillation^7^.

Asia, home to over 60% of the world’s population, has the highest incidence rate of stroke globally^9^. Malaysia’s healthcare system comprises the public and private sectors^10^. Major speciality, minor specialist, and district hospitals are all parts of Malaysia’s public hospital system, overseen by the Ministry of Health (MOH)^11^. In Malaysia, public hospital wards are often separated into three categories (1st class, 2nd class, and 3rd class, mainly based on room cost)^12^. Most acute stroke cases are treated in third-class wards, which account for 86.4% of all the beds. The government heavily subsidizes the beds in the third-class ward, and Malaysians only pay a meager MYR3.00 per day for basic ward fees^13^.

The cost of acute care for ischemic stroke is a major concern for healthcare systems, as it places a substantial financial burden on the health system. Understanding the drivers of the stroke costs across the various phases of care is essential for informing policy decisions and could lead to improved resource allocation and quality of care. Existing evidence has shown variable estimates of the medical expenses due to in-part heterogeneity in the stroke population^14^. However, there is no information on the inpatient medical cost of a first-ever ischemic stroke in Malaysia. Therefore, this study aimed to estimate the inpatient direct medical cost of patients with first-ever ischemic stroke in Malaysia.

## Methods

### Study design

The study was a retrospective study that estimated the inpatient direct medical costs of first-ever ischemic stroke. The study utilized the medical records of the patients managed at Hospital Sultanah Nur Zahirah, Terengganu, Malaysia (HSNZ), and enrolled in the Malaysian National Stroke Registry (NSR) from 2009 to 2020. The details of the NSR have been described previously^15,16^.

## Study population

The study included all adult patients aged 18 years and above with a confirmed diagnosis of first-ever ischemic stroke managed at HSNZ and enrolled into the NSR. The HSNZ was a representative hospital that serves as one of Malaysia’s major stroke referral centers, contributing to the NSR since its inception in 2009, with more patients^15^. In addition, all the NSR-participating hospitals owned by the Malaysian government are governed by the same operational guidelines, as described elsewhere^17,18^. From the NSR, a total of 899 patients were identified as first-ever ischemic stroke, recruited from several participating clinical sites. Out of the 899 patients, only 122 with first-ever ischemic stroke managed at HSNZ were included in the final analysis.

## Sample size Estimation

The sample size for this study was calculated using a standard formula for descriptive cost-of-illness studies^19^. With a 95% confidence interval (Z = 1.96), an expected prevalence (P) of 76% based on prior research^20^and a margin of error (d) of 5%, the minimum required sample size was approximately 120 participants.

## Cost analysis

The study involved a prevalence-based and direct medical micro-costing approach from the healthcare provider’s perspective (Ministry of Health, Malaysia). Micro-costing is a bottom-up methodology based on the services provided to the patients during the care period. Direct medical costs are expenses related to the use of medical resources beginning with patient hospital admission (bedroom), radiology investigations (e.g., head CT scan, electrocardiogram, carotid ultrasound, and thoracic X-ray), laboratory tests (e.g., full blood count, urea creatinine and electrolyte (UCr&E), blood glucose, cholesterol, liver function tests and albumin/bilirubin) and medications (e.g., alteplase, antiplatelets, anticoagulants, antihypertensive, antidiabetic and antihyperlipidemic).

### Statistical analysis

Data were imported into IBM SPSS Statistics for Windows, version 26.0 (IBM Corp., Armonk, NY, USA) for analysis. Missing data were addressed using multiple imputation methods^21^. Categorical variables were summarized as frequencies and percentages, while continuous variables were presented as mean (standard deviation) or median (interquartile range, IQR), depending on distribution. Normality was assessed using the Kolmogorov-Smirnov test (*p* > 0.05 indicated data as normaly distributed) and visual inspection of the histogram. Differences between continuous variables were tested using the Chi-square or Fisher’s exact tests (the latter for expected frequencies below 10). In contrast, differences between two or more groups with continuous variables were analyzed using the Mann-Whitney U and Kruskal-Wallis H tests, respectively. Statistical significance was set at *p* ≤ 0.05.

## Results

### Characteristics of patients with first-ever ischemic stroke

The study included a total of 122 patients with first-ever ischemic stroke. The average age ± standard deviation (SD), was 61.0 ± 10.9 years, with the majority being 60 years or older (61.5%). About half of the patients were males (50.8%). The patients were classified according to the Oxfordshire Community Stroke Project (OCSP) as mostly lacunar Infarct (LACI) (59.8%). The average (± SD) length of hospital stay (LOS) was 4.5 ± 3.5 days (range, 1–22 days), with the majority staying ≤ 3 days (52.5%). The common risk factors were hypertension (71.3%), diabetes (41.0%), and hyperlipidemia (17.2%). Most patients had a functional disability defined as poor functional status (mRS ≥ 3) (63.9%), with an average mRS score of 3.0 (± 1.1). The primary outcome measured at the end of this phase was the direct medical cost of inpatient care. The overall average inpatient direct medical cost (± SD) was calculated as MYR 1,970.7 ± 1,385.8 per patient. (Table 1).

**Table 1:** Characteristics of patients with first-ever ischemic stroke.

Variables	*n* (%)
**Age**	61.0 (10.9)^a^
< 60 years	47 (38.5)
≥ 60 years	75 (61.5)
**Gender**	
Male	62 (50.8)
Female	60 (49.2)
**Stroke subtype**	
TACI	12 (9.8)
PACI	21 (17.2)
LACI	73 (59.8)
POCI	16 (13.1)
**mRS scores**	3.0 (1.1)^a^
Favorable status (0–2)	44 (36.1)
Unfavorable status (3–5)	78 (63.9)
**Length of stay**	4.5 (3.5)^a, b^
≤ 3 days	64 (52.5)
4–7 days	43 (35.2)
> 7 days	15 (12.3)
**Risk factors**	
Hypertension	87 (71.3)
Diabetes	50 (41.0)
Hyperlipidemia	21 (17.2)
Ischemic Heart Disease	11 (9.0)
Others	16 (13.1)
**Overall cost**	MYR1970.7 (1385.8)^a^

**Keys**: ^a^: mean ± standard deviation (SD); ^b^: range, 1–22 days; ; TACI: Total Anterior Circulation Infarct; PACI: Partial Anterior Circulation Infarct; LACI: Lacunar Infarct; POCI: Posterior Circulation Infarct; OCSP: Oxfordshire Community Stroke Project; SD: Standard deviation; mRS: modified Rankin Scale; Cost denomination: MYR: Malaysian Ringgit; *N* = 122

## Comparison of patients’ characteristics and medical costs

The direct medical cost of first-ever ischemic stroke was significantly different among the OCSP groups (*p* < 0.001), with PACI having the highest average (±SD) cost (MYR 2753.4 ± 2268.6) and LACI the lowest (MYR 1696.6 ± 1158.9). In addition, the medical cost was significantly higher in patients with unfavorable functional status (MYR 2235.7 ± 1636.4, *p* = 0.002), highest in patients with LOS >7 days (MYR 4828.1 ± 2238.2,  *p* < 0.001), and lower in patients with risk factors (MYR 1922.1 ± 1416.4, *p* = 0.023). There was no significant difference in the medical cost based on gender (*p* = 0.910) or age (*p* = 0.087). (Table 2).

**Table 2:** Comparison of patients’ characteristics and medical costs.

Variables	Mean cost (SD), MYR	*p*-value
Gender		
Male	1929.1 (1194.9)	0.910
Female	2013.7 (1568.1)	
**Age**		
< 60 years	1762.1 (964.8)	0.087
≥ 60 years	2101.4 (1586.4)	
**OCSP classification**		
TACI	2168.6 (746.8)	< 0.001^#^
PACI	2753.4 (2268.6)	
LACI	1696.6 (1158.9)	
POCI	2045.8 (716.6)	
**Functional status (mRS)**		
Favorable (0–2)	1500.9 (505.8)	0.002*
Unfavorable (3–5)	2235.7 (1636.4)	
**Length of stay**		
≤ 3 days	1301.3 (245.6)	< 0.001^#^
4–7 days	1970.3 (417.0)	
> 7 days	4828.1 (2238.2)	
**Risk factors (Yes/No)**		
Risk Factors	1922.1 (1416.4) vs. 2141.9 (1282.7)	0.023*
Hypertension	1956.9 (1460.2) vs. 2005.0 (1200.0)	0.203
Diabetes	1850.4 (1249.9) vs. 2054.2 (1475.7)	0.132
Hyperlipidemia	1737.9 (591.2) vs. 2019.1 (1496.7)	0.150
Ischemic Heart Disease	2413.3 (2010.6) vs. 1926.8 (1312.8)	0.103
Others	1457.2 (568.9) vs. 2002.5 (1469.4)	0.138

**Keys**: Cost Denomination: MYR: Malaysian Ringgit); TACI: Total Anterior Circulation Infarct; PACI: Partial Anterior Circulation Infarct; LACI: Lacunar Infarct; POCI: Posterior Circulation Infarct; OCSP: Oxfordshire Community Stroke Project; SD: Standard Deviation; *: Mann-Whitney U Test; ^#^: Statistical Significance at *p* ≤ 0.05 using Kruskal-Wallis H Test.

### Comparisons of medical costs based on patients’ risk factors

The overall average inpatient direct medical cost of the patients with first-ever ischemic stroke was MYR 1,970.7 ± 1,385.8. The overall cost was primarily contributed by the hospital admission fees (MYR 754.6 ± 632.2) and radiology investigations (MYR 794.0 ± 284.4). Other cost components includelaboratory tests (MYR 276.7 ± 234.7) and medications (MYR 145.0 ± 558.0). Laboratory investigation costs were significantly higher in patients with risk factors (*p* = 0.040). (Table 3).

**Table 3:** Comparisons of medical costs based on patients’ risk factors.

Components	Mean cost (SD), MYR			*p*-value
	Overall	Risk factors		
		Yes	No	
**Admission**	**754.6 (632.2)**	**734.7 (632.2)**	**824.4 (639.3)**	**0.465**
**Radiology**	**794.0 (284.4)**	**771.6 (279.9)**	**872.8 (291.1)**	**0.050**
CT scan, head	606.8 (224.1)	596.8 (224.7)	553.7 (222.4)	
Electrocardiogram	37.3 (19.8)	37.4 (20.4)	36.7 (20.4)	
Carotid ultrasound	67.6 (126.6)	64.7 (125.0)	77.8 (134.0)	
X-Ray, thoracic	82.4 (103.4)	72.6 (98.3)	116.7 (115.2)	
**Laboratory**	**276.7 (234.7)**	**268.6 (250.2)**	**305.2 (169.9)**	0.040*
Full blood count	89.9 (107.9)	86.5 (113.9)	102.1 (84.3)	
UCr&E	45.3 (64.4)	43.7 (68.5)	51.1 (47.8)	
Blood glucose	45.6 (33.0)	48.0 (33.8)	37.0 (28.7)	
Cholesterol	41.6 (14.0)	41.7 (14.2)	41.5 (13.5)	
Liver function tests	21.6 (42.1)	20.3 (45.5)	25.9 (27.1)	
Albumin/bilirubin	9.5 (20.0)	8.2 (20.1)	14.1 (19.1)	
Others	23.2 (40.8)	20.2 (37.2)	33.5 (51.0)	
**Medication**	**145.0 (558.0)**	**147.1 (598.8)**	**139.5 (391.0)**	**0.197**
Thrombolytics	79.2 (525.9)	82.9 (568.6)	66.0 (343.1)	
Antiplatelets	0.6 (0.8)	0.6 (0.4)	0.8 (1.5)	
Anticoagulants	19.0 (48.6)	17.2 (45.8)	25.4 (57.9)	
Antihypertensive	1.5 (6.6)	1.7 (7.3)	1.2 (2.7)	
Antidiabetic	6.4 (16.1)	6.5 (13.1)	6.3 (24.0)	
Antihyperlipidemic	0.7 (2.1)	0.8 (2.4)	0.3 (0.4)	
Others	37.9 (110.3)	37.5 (116.4)	39.3 (87.3)	
**Overall average cost**	**1970.7 (1385.8)**	**1922.1 (1416.4)**	**2141.9 (1282.7)**	**0.203**

**Keys**: Cost denomination: MYR, Malaysian ringgit; CT: Computerized tomography; SD: Standard deviation; UCr&E: Urea, Creatinine & Electrolytes; *: Statistical Significance at *p* ≤ 0.05 using Mann-Whitney-U Test.

### Comparisons of medical costs based on patients’ functional status

The overall average direct medical cost (*p* = 0.002), hospital admission cost (*p* = 0.001) and medications cost (*p* < 0.001) were significantly higher among patients with functional disabilities than those with favorable functional status. There was no significant difference in the cost of radiology investigations (*p* = 0.108) and laboratory services (*p* = 0.133) between patients based on their functional status (Table 4).

**Table 4:** Comparisons of cost components based on functional status.

Components	Mean cost (SD), MYR		*p*-values
	Functional status (mRS)		
	Favorable	Unfavorable	
**Admissions**	**515.9 (273.4)**	**889.2 (731.8)**	0.001*
**Radiology**	**740.7 (217.3)**	**824.1 (313.3)**	**0.108**
CT scan, head	572.7 (152.7)	626.0 (254.7)	
Electrocardiogram	37.1 (19.3)	34.0 (19.4)	
Carotid ultrasound	58.0 (126.2)	73.1 (127.3)	
X-Ray, thoracic	67.1 (93.4)	91.0 (108.3)	
**Laboratory**	**224.9 (88.1)**	**305.9 (282.5)**	**0.133**
Full blood count	66.3 (32.6)	103.3 (131.2)	
UCr&E	26.3 (40.6)	49.1 (74.6)	
Glucose	39.1 (28.9)	49.2 (34.7)	
Cholesterol	41.8 (12.1)	41.5 (15.1)	
Liver function tests	13.6 (23.1)	26.0 (49.3)	
Albumin/Bilirubin	8.2 (16.3)	10.3 (21.8)	
Others	17.3 (24.2)	26.5 (47.5)	
**Medications**	**19.5 (40.3)**	**216.5 (688.7)**	< 0.001*
Thrombolytics	0 (0)	123.8 (654.9)	
Antiplatelets	0.5 (0.3)	0.8 (1.0)	
Anticoagulants	1.7 (8.7)	28.8 (58.4)	
Antihypertensive	2.6 (10.0)	0.9 (3.3)	
Antidiabetic	4.1 (11.3)	7.7 (18.1)	
Antihyperlipidemic	0.7 (2.5)	0.7 (1.8)	
Others	9.9 (25.7)	53.7 (134.3)	
**Overall average cost**	**1500.9 (505.8)**	**2235.7 (1636.4)**	0.002*

Cost Denomination: MYR, Malaysian Ringgit; CT: Computerized Tomography; mRS: Modified Rankin Scale; UCr&E: Urea, Creatinine & Electrolytes; SD: Standard Deviation; mRS 0–2: Favorable (good) functional status; mRS 3–5: Unfavorable (poor) functional status; *: Statistical Significance at *p* ≤ 0.05 using Mann-Whitney U Test.

## Discussion

This study used the prevalence-based approach to estimate the direct medical cost of inpatient care for patients with first-ever ischemic stroke in in Malaysia. The prevalence approach provides more accurate estimates by assessing the current economic burden of a disease, rather than projected costs, and is often used in conditions that result in long-term consequences, such as stroke^22^. In this study, the direct costs of inpatient care following a first-ever ischemic stroke were substantial. The costs were found to be associated with the length of stay, clinical characteristics, stroke types and components of the stroke care process, including admission, radiology, laboratory, and medications. The first step in stroke care is early identification of patients with stroke and triage to centers capable of delivering the appropriate treatment as fast as possible^23^. The present study is the first to provide estimates of the distribution of the inpatient direct medical costs across the different stroke care process components in Malaysia.

In this study, the average age of the patients was 61.0 (± 10.9) years, with most being 60 years or older. Similar studies conducted in Malaysia reported a comparable average age of patients with ischemic stroke of 59.0 to 62.8 years^24–27^. The prevalence of older patients with stroke varies among countries. Comparable results were shown in neighbouring countries to Malaysia, like Singapore^28,29^Thailand^30–32^and Indonesia^33^and slightly higher in China^34–36^ and Taiwan^37,38^. However, studies from Western countries, particularly the US, UK, and EU, reported a much older age of 70 and above^39–42^. Age is the most significant non-modifiable risk factor for ischemic stroke, and older stroke patients experience worse functional recovery, higher mortality, and morbidity^43^. The distribution of ischemic stroke subtypes according to the OCSP classification was primarily LACI, accounting for 59.8%. The higher number of patients with LACI and the lower number with TACI stroke subtypes are consistent with previous studies^44,45^. Hypertension (71.3%) and diabetes (41.0%) were the most common risk factors, followed by hyperlipidemia (17.2%), identified in this study. Studies from other countries showed comparable results, with hypertension, diabetes, and hyperlipidemia consistently predominant risk factors of acute stroke^46–48^. Similar risk factors were prevalent in many Asian studies, including studies from northern China and Southeast Asia^30,49,50^. Identifying and targeting risk groups would be relevant for stroke prevention or improved clinical management. The functional outcomes, as measured by the mRS, identified most patients with an unfavorable status (mRS ≥ 3, 63.9%).

The average cost of inpatient care for patients with first-ever ischemic stroke was found to be substantial. The most significant cost component was the cost of hospital admission, which accounted for approximately 46% of the total cost of the stroke care. The higher cost of admission could be attributed to the prolonged length of hospital stay during the acute phase of the stroke care, and associated hospital bedroom costs. Laboratory investigations and imaging studies were the next most significant medical cost components, accounting for approximately 25% and 16% of the total cost of the stroke care, respectively. These findings highlight the importance of optimizing resource utilization in stroke care, such as reducing unnecessary laboratory investigations and imaging studies to minimize costs, as in other studies^51,52^.

Remarkably, the daily cumulative costs applied to all four care components (admission, radiology, laboratory, and medications) are for those with limited utility. The small percentage of total costs due to radiology investigations suggests they were used less frequently. The mean LOS in our study was short, although longer than those reported in other studies^52^. Stroke severity and discharge destination were the most significant factors influencing LOS. Although it is easy to explain a direct relationship between the severity of deficits and the time spent in the hospital, the link between LOS and discharge destination may have more than one reason. On the other hand, it may be that some destinations, such as nursing homes and rehabilitation centers, have long waiting lists and, therefore, require a LOS and higher costs. These observations suggest that more institutions, such as home assistance services or rehabilitation wards, may need to care for patients after the acute phase.

In this study, functional disability was measured using the mRS score, with a score of 3 or greater indicating disability. Interestingly, the study found that clinical variables had a relatively limited impact on the additional costs incurred by stroke patients, likely due to the standardized protocol used, which does not heavily depend on stroke severity. However, the study did find an inverse relationship between ancillary costs and risk factors, which may be explained by the need for more investigations in younger patients to identify any unusual causes of stroke or to define better the vascular status and extent of cerebral damage through imaging studies such as angiography and MRI. The availability of such facilities may be an essential factor in determining the overall cost of hospitalization for ischemic stroke patients. The cost of medications may be higher. A different organizational approach may be necessary to admit and treat patients as early as possible, potentially reducing the length of stay and need for further care. However, the net impact on costs is difficult to predict. Overall, the study’s bottom-up approach, which calculates the cost of each patient based on the actual costs of personnel and examinations, provides valuable insights into the components of hospital costs and may suggest ways to improve the organization of diagnostic and treatment pathways while maintaining high-quality care. Further research is needed to explore the impact of other factors on the cost of stroke care and to develop strategies to improve stroke prevention and treatment while minimizing costs.

The study’s limitations include its relatively smaller sample size, reliance on administrative registry data from a single hospital, limited scope of cost estimation, and lack of consideration of indirect and long-term costs. This study did not account for indirect costs, such as loss of productivity and caregiver burden, which may have resulted in an underestimate of the total economic burden of the stroke cohort considered. Future research should include larger sample sizes and indirect costs to allow for more comprehensive evidence on the economic burden of first-ever acute ischemic stroke in Malaysia. The effects of inflation over the study period were not considered in the cost analysis, which may affect the comparability of costs across years. Finally, non-parametric tests were prioritized for hypothesis testing due to skewed data; mean (SD) are presented descriptively and should be interpreted with caution for highly non-normal distributions.

## Conclusion

The present study demonstrates that the inpatient direct medical costs of first-ever ischemic stroke in Malaysia were substantial and influenced by stroke subtypes, length of stay, risk factors, and functional status. The findings of this study can inform policy decisions aimed at improving acute stroke care. Strategies to reduce the length of stay, improve the management of comorbidities, and functional status can potentially reduce the cost burden of stroke care. Furthermore, the findings of this study can inform interventions to ease the economic burden of stroke on patients, their families, and the healthcare system.

## Data Availability

The datasets generated during and/or analyzed during the current study are available from the corresponding author on reasonable request.
